# Pandemic preparedness: perceptions of vulnerable migrants in Thailand towards WHO-recommended non-pharmaceutical interventions: a cross-sectional study

**DOI:** 10.1186/1471-2458-14-665

**Published:** 2014-06-28

**Authors:** Jason Hickey, Anita J Gagnon, Nigoon Jitthai

**Affiliations:** 1 University of Calgary in Qatar, PO Box 23133, Doha, Qatar; 2 McGill University Health Centre, 2155 Guy St. #400-09, H3H 2R9 Montreal, QC, Canada; 3 USAID Regional Development Mission for Asia, 25F, Athenee Tower, 63 Wireless Road, 10330 Pathumwan, Bangkok, Thailand

**Keywords:** Influenza, Pandemic preparedness, Disease prevention, Vulnerable migrants, Non-pharmaceutical interventions, Public health

## Abstract

**Background:**

Non-pharmaceutical interventions (NPIs) constituted the principal public health response to the previous influenza A (H1N1) 2009 pandemic and are one key area of ongoing preparation for future pandemics. Thailand is an important point of focus in terms of global pandemic preparedness and response due to its role as the major transportation hub for Southeast Asia, the endemic presence of multiple types of influenza, and its role as a major receiving country for migrants. Our aim was to collect information about vulnerable migrants’ perceptions of and ability to implement NPIs proposed by the WHO. We hope that this information will help us to gauge the capacity of this population to engage in pandemic preparedness and response efforts, and to identify potential barriers to NPI effectiveness.

**Methods:**

A cross-sectional survey was performed. The study was conducted during the influenza H1N1 2009 pandemic and included 801 migrant participants living in border areas thought to be high risk by the Thailand Ministry of Public Health. Data were collected by Migrant Community Health Workers using a 201-item interviewer-assisted questionnaire. Univariate descriptive analyses were conducted.

**Results:**

With the exception of border measures, to which nearly all participants reported they would be adherent, attitudes towards recommended NPIs were generally negative or uncertain. Other potential barriers to NPI implementation include limited experience applying these interventions (e.g., using a thermometer, wearing a face mask) and inadequate hand washing and household disinfection practices.

**Conclusions:**

Negative or ambivalent attitudes towards NPIs combined with other barriers identified suggest that vulnerable migrants in Thailand have a limited capacity to participate in pandemic preparedness efforts. This limited capacity likely puts migrants at risk of propagating the spread of a pandemic virus. Coordinated risk communication and public education are potential strategies that may reduce barriers to individual NPI implementation.

## Background

We have recently seen the emergence of two new pathogens that are being closely monitored by public health agencies due to their pandemic potential. One is a new Avian Influenza A (H7N9) virus in China that has developed the ability to transmit from human-to-human. The other is the Middle East respiratory syndrome coronavirus with mortality rates above 50%. Discovery of these pathogens highlight the importance for public health officials worldwide to continue pandemic preparedness efforts. One key strategy employed during the previous influenza A (H1N1) 2009 pandemic was the use of non-pharmaceutical interventions (NPIs). Examining and enhancing individuals’ attitudes towards NPIs is one important area of preparation for a new pandemic. This paper presents data collected during the 2009 pandemic about the perceptions of migrants in Thailand towards NPIs and their ability to implement these NPIs. Many potential barriers were identified.

Non-pharmaceutical interventions such as personal hygiene, cough etiquette, social distancing and border measures constituted the principal tools employed in global efforts to mitigate the influenza A (H1N1) 2009 pandemic. NPIs were heavily relied upon during the early stages of the pandemic to slow disease transmission, while work was undertaken to understand the virus and develop a vaccine [1]. Antivirals were available in many countries but potential development of resistance presented a major concern [2], highlighting the importance of NPIs to reduce reliance on antivirals. During future pandemics it is likely that NPIs will again constitute our principal set of tools to reduce transmission, gain time to put response measures into place and work towards vaccine development.

Thailand is an important point of focus in terms of global pandemic preparedness and response due to its role as the major transportation hub for Southeast Asia, the endemic presence of multiple types of influenza, and its role as a major receiving country for migrants. Certain groups of migrants may be particularly vulnerable to pandemic influenza due to traditions in raising poultry and swine, poor personal hygiene and sanitation, low levels of health knowledge and awareness, and limited access to health care [3-6]. Some migrant populations in Thailand share these characteristics [7]. Furthermore, migrants’ proximity to international borders may increase likelihood of cross-border disease communication and occurrence of future pandemics [8].

Thailand’s Ministry of Public Health (MOPH) works collaboratively with the International Organization for Migration (IOM) to improve the health and well-being of potentially vulnerable migrant groups. IOM’s work is focused in ‘priority provinces’ that have been designated as such, based on the high concentration of migrants and frequency of cross-border communication (i.e., movement of individuals and goods). The proportions of migrants compared to Thai people living in border areas vary widely and depend on how one defines “migrants”. In this context, we define it as any individuals that do not have a Thai citizenship, regardless of their places of birth or immigration status. The two studied provinces are among the top five in the country regarding the size of migrant populations. There are an estimated two and a half million migrants providing unskilled labour in Thailand, nearly one and a half million being undocumented [9].

Thailand’s past experience with Avian Influenza outbreaks meant that pandemic preparedness guidelines and policies had been put into place prior to the influenza A (H1N1) 2009 pandemic [3]. NPIs, including hand hygiene, social distancing, face masks and border measures were all included in the guidelines, were widely promoted and implemented during the pandemic [10]. Thailand’s first case of A (H1N1) pdm09 was reported in early May, 2009. In total, 47,433 confirmed cases and 347 resultant deaths were reported [10]. Failure of NPIs to prevent widespread transmission of influenza A (H1N1) pdm09 highlights the need to identify factors that may reduce the effectiveness of NPIs during a pandemic.

Several studies have demonstrated that individual characteristics of non-migrant populations are closely linked to NPI adherence [11,12]. An anonymous telephone survey of 999 adults in Hong Kong revealed perceived efficacy of hand washing and face mask use to be ‘quite effective’ in nearly 70% of respondents [12]. Positive perceptions were linked to higher levels of hand-washing and face mask use. Another telephone survey of 1000 individuals in England, Scotland and Wales found that perceived efficacy toward disinfection measures and hand washing were quite high (more than 80% answered ‘tend to agree’ or ‘agree’) but was lower towards social distancing, face mask use, and avoiding hospitals [11]. This study also found an association between perception and NPI adherence. Both studies highlight the importance of assessing and addressing individuals’ perceptions of NPIs.

During the influenza A (H1N1) 2009 pandemic, IOM and the McGill University School of Nursing undertook a study to identify influenza knowledge, attitudes, and practices among migrants in Thailand. One assumption guiding a subset of questionnaire development was that it is ultimately an individual decision to adhere to NPI recommendations. In addition, the most recent revision of the WHO guidance document on pandemic preparedness incorporates a more explicit and active role for communities, individuals and families [13]. The guidance document suggests that respiratory hygiene, hand washing and voluntary isolation of cases may help limit the spread of influenza, but it does not address individuals’ willingness or ability to undertake these actions.

Our aim was to collect information about vulnerable migrants’ perceptions of and ability to implement NPIs proposed by the WHO. We hope that this information will help us to gauge the capacity of individuals within the vulnerable migrant community to participate in pandemic preparedness and response efforts, and to identify potential barriers to NPI effectiveness.

## Methods

### Study population

Study participants (n = 801) were recruited from two provinces in Northern Thailand adjacent to the Myanmar and Laos borders, Chiang Rai and Tak. Participants were sampled from all known migrant-populated communities within these provinces. First, maps created by Migrant Community Health Workers (MCHWs) during a previous IOM/MOPH project that outline the number and locations of households within each community were used to randomly select households. The number of households chosen from each map was based on the proportion of migrants in that village compared to the rest of the province. A web application, research randomizer [14] was used to randomly generate the specific household numbers to sample. Second, data collectors approached members of the selected households and requested a volunteer from each household to complete the survey. The decision to seek volunteers from each household was made after extensive consultation with IOM and MCHWs as the most ethically and culturally appropriate method in this context. Consideration was given to sampling the household heads, but it was felt that this would have turned our sample into a predominately male one and put unintended pressure on this person to participate. Random selection of individuals was also considered, but it was likely that this method would have been culturally offensive to some groups. Data collectors attempted to recruit equal numbers of male and female migrants by requesting a female volunteer from the first household, a male from the second household, a female from the third, and so forth. It was requested that volunteers be between the ages of 18 and 65 without any known psychological disability that would prevent them from completing the survey.

### Data collection and survey method

Interviewer-assisted questionnaires were administered between September and November, 2009 by MCHWs employed by the MOPH. MCHWs travelled to the communities where they are known to the migrant population and familiar with the culture and language(s) spoken. Participants spoke a range of ethnic languages so MCHWs were selected who had fluency in one or more of these languages. Interviews were conducted in the participant’s primary language, in Thai, or in a mixture of both, depending on participant’s preferences and language abilities. Most MCHWs had been involved in previous health promotion activities and data collection and were familiar with negotiating this communication process. MCHWs were responsible for describing the purpose of the study, the risks and benefits of participation, obtaining informed consent, and collecting data. All MCHWs received training on research methodology, survey administration and research ethics prior to data collection. This study received ethical approval from the McGill University Research Ethics Committee and underwent review by IOM for cultural appropriateness prior to recruitment.

The subset of data presented in this paper contains information on socio-demographic factors and perceptions and practices relating to the following WHO-defined categories of NPIs: measures to reduce risk that cases transmit infection, measures to reduce risk that contacts transmit infection, measures to increase social distance, disinfection measures, and border measures [15].

A 201-item interviewer-assisted questionnaire was used to collect data. This instrument was revised from a previous IOM influenza questionnaire to incorporate key components of the WHO’s Global Influenza Preparedness Plan, Pandemic Preparedness Checklist [15,16] and related themes from the literature [4,6,17-23]. Pandemic preparedness ‘experts’ working with IOM provided their feedback on the revised version and it was adjusted accordingly. It was then translated from English into Thai using two independent translation services. Each translation was subjected to review by one author (NJ) and the one judged to be superior was blind back-translated [24]. The back-translation was compared to the original English version and necessary adjustments were made. MCHWs provided further feedback on the questionnaire during training sessions and revisions were made to ensure cultural appropriateness. The questionnaire was then piloted with non-study participants and revised one final time for clarity. Interpretation of the Thai questionnaire into the primary languages of the participants was rehearsed extensively during training to ensure accuracy and equivalency between MCHWs. The subset of results reported in this paper include, socio-demographic variables and measures to reduce risk of disease transmission. These data were collected using closed-ended questions.

One to two days of data collection were observed in each province by one author (JH) to ensure quality and consistency of questionnaire administration. Additionally, each data collector’s first three interviews were audio-recorded and reviewed for consistency. MCHWs were required to review completed questionnaires for missing or unclear responses and to resolve these before leaving the participant’s home. Questionnaires were reviewed for completeness and logical data checks were made prior to computer data entry. Questions arising were resolved with the MCHW. The first 20 questionnaires from each province that were entered by the data-entry clerk were re-entered by one author (JH) in their entirety to assess for errors. If errors were discovered, feedback was given to the data-entry clerk and/or MCHWs, the error corrected, and the next 20 records examined similarly. A 5% random sample of questionnaires was later re-entered to confirm data quality.

### Data analysis

Univariate descriptive analyses were conducted and frequency tables were created using Microsoft Excel. Questionnaires with incomplete response sets for this data subset (n = 28) were omitted from analysis. Data from both provinces were combined to provide a broad assessment of migrants’ practices and perceptions that could be used by IOM and the MOPH to implement policies and programs at a national level. The response category of ‘unsure/declined to answer’ was included as a valid response for the purposes of data analysis. Previous work at IOM has shown that this category of response is typically high among this population as migrants are careful to avoid giving answers that may be viewed negatively by health/governmental authorities. It is an important response item in terms of analysis because it helps us gauge migrant’s uncertainty about the survey questions as well as their comfort level with participation. Results were summarized within each category of NPI.

## Results

### Socio-demographic

Data collectors were able to enroll a volunteer from each household selected for sampling. A total of 773 participants were included in analyses, 373 from Tak Province and 400 from Chiang Rai Province. Forty-nine percent were between 18 to 35 years of age. Fifty-one percent were female. Education levels were low, 46% having no formal education, and only 13% having completed more than six years. Seventy-five percent were able to have at least a basic conversation in Thai. Over one in five were unemployed and of those employed, the most common job reported was daily labourer (40%). Median family income was USD94 (THB3000) per month and supported an average household of 4.6 family members (SD 2.32, range 0-15).

### Measures to reduce risk that cases transmit infection

Only 21% of participants responded that they would agree to stay inside their homes if sick with an influenza-like illness during an outbreak. The majority (73%) were unsure what action they would take or declined to answer the question. Hospital (43%) was the preferred location for confinement, followed by the home (29%).

One quarter of respondents had used face masks in the past when sick. Slightly less (24%) said they would agree to use a face mask if sick in the future and over half (53%) said they would not wear a face mask. Only one third believed that wearing a mask could prevent the transmission of illness. Further results are presented in Table 1.

**Table 1:** Measures to reduce risk that cases transmit infection (n = 773)

**Measure**	**%**
Confinement	
Would agree to stay inside home if sick	
Yes	21%
No	6%
Unsure/Refused to answer	73%
Preferred location of isolation	
Hospital	43%
Home	29%
School	0%
Other location	2%
Unsure/Refused to answer	25%
Face Masks	
Has ever used a face mask when sick with ILI	
Yes	25%
No	55%
Unsure/Refused to answer	20%
Would agree to wear face mask if sick	
Yes	24%
No	53%
Unsure/Refused to answer	23%
Believes that using a face mask could prevent transmission of illness	
Yes	33%
No	25%
Unsure/Refused to answer	42%
Would agree to wear face mask after exposure to someone who is sick	
Yes	12%
No	67%
Unsure/Refused to answer	21%
Would agree to wear face mask while waiting at a health facility	
Yes	8%
No	70%
Unsure/Refused to answer	22%

### Measures to reduce risk that contacts transmit infection

The majority of respondents (77%) said that if they were sick with an influenza-like illness they would agree to tell health authorities so that contacts could be located. Most (70%) would feel more comfortable giving this information to a MCHW. Less than half (48%) reported that they would be able to check their own temperature at home; the most common barriers to doing so were not owning a thermometer (30%) and not knowing how (28%).

Participants were given a scenario in which they had been in contact with someone sick with influenza. In response, just over half (55%) would agree to take preventative medicine and avoid travelling to places with no signs of outbreak.

When given a hypothetical of a disease outbreak or pandemic, nearly half (46%) said they would remain in their community. Some said they would move to another community in Thailand (11%) and a few said they would move back to their home country (3%). If official border crossings were closed, some participants would travel through other routes (12%). Just under half of respondents (44%) agreed that banning cross-border travel during a pandemic could help prevent the spread of disease. Further results are presented in Table 2.

**Table 2:** Measures to reduce risk that contacts transmit infection (n = 773)

**Measure**	**%**
Tracing and follow-up of contacts	
Would inform health authorities of ILI so contacts could be located	
Yes	77%
No	1%
Unsure/Refused to answer	21%
Would feel more comfortable giving information to MCHW/V*	
Yes	70%
No	4%
Unsure/Refused to answer	26%
Self-health monitoring	
Able to check temperature at home prior to travel	
Yes	48%
No	28%
Unsure/Refused to answer	23%
Barriers to checking own temperature	
Don't own a thermometer	30%
Don't know how	28%
Other reasons	7%
Voluntary quarantine of healthy contacts	
*After being in contact with someone sick with influenza:*	
Would take preventative medication	
Yes	55%
No	33%
Unsure/Refused to answer	12%
Would avoid travelling to places with no new cases	
Yes	54%
No	34%
Unsure/Refused to answer	12%
Would tell health authorities if they became sick	
Yes	52%
No	29%
Unsure/Refused to answer	19%
Advise contacts to defer travel to unaffected areas	
Potential action if outbreak/pandemic in community	
Do nothing	46%
Move to another community in Thailand	11%
Return to home country	3%
Other	10%
Unsure/Refused to answer	29%
Believes that banning cross-border travel could prevent disease spread	
Yes	44%
No	7%
Unsure/Refused to answer	50%
If borders were closed during pandemic:	
Would postpone/cancel trip	60%
Would travel through other routes	12%
Other	2%
Unsure/Refused to answer	26%

*MHCW/V = Migrant Community Health Worker/Volunteer.

### Measures to increase social distance

Respondents were asked whether they thought various social distancing measures would be effective at reducing the spread of illness during periods of disease outbreak. Avoiding gatherings of five or more people received the most positive responses (54%), followed by avoiding places of entertainment (44%), avoiding department stores, supermarkets and minimarts (39%), avoiding restaurants (38%), limiting contact with family and friends (36%), avoiding public transportation (36%), keeping children from school (34%), avoiding the workplace (31%), avoiding the hospital (31%), and avoiding the public health centre (28%). Further results are presented in Table 3.

**Table 3:** Measures to increase social distance (n = 773)

**Measure**	**%**
Believes following interventions can reduce spread of illness during outbreak:	
Avoiding gatherings of more than five people	
Yes	54%
No	29%
Unsure/Refused to answer	16%
Avoid places of entertainment (e.g., karaoke bars, pool house, etc.)	
Yes	44%
No	34%
Unsure/Refused to answer	22%
Avoiding department stores, supermarket, minimart	
Yes	39%
No	36%
Unsure/Refused to answer	25%
Avoiding restaurants	
Yes	38%
No	38%
Unsure/Refused to answer	24%
Limiting contact with family/friends	
Yes	36%
No	41%
Unsure/Refused to answer	22%
Avoiding public transportation	
Yes	36%
No	46%
Unsure/Refused to answer	19%
Keeping children from school	
Yes	34%
No	49%
Unsure/Refused to answer	17%
Avoiding Workplace	
Yes	31%
No	46%
Unsure/Refused to answer	24%
Avoiding the hospital	
Yes	31%
No	48%
Unsure/Refused to answer	20%
Avoiding public health centre	
Yes	28%
No	52%
Unsure/Refused to answer	20%

### Disinfection measures

Less than half of all respondents (46%) said they would increase hand washing frequency during an outbreak or pandemic. Among those who would increase hand washing 50% do not use any form of soap. Less than half (45%) agreed that hand washing can reduce the transmission of illness during periods of disease outbreak. Like hand washing, only 48% of those who would increase disinfection frequency during a pandemic (51%) would use some form of soap. Further results are presented in Table 4.

**Table 4:** Disinfection measures (n = 773)

**Measure**	**%**
Believes increased hand washing can reduce spread of illness during outbreak	
Yes	45%
No	5%
Unsure/Refused to answer	50%
Would increase hand washing frequency during an outbreak or pandemic	
Yes	46%
No	16%
Unsure/Refused to answer	38%
Materials routinely used to wash hands	
Clean water only	59%
Clean water and soap, detergent or dishwashing liquid	38%
Unsure/Refused to answer	1%
Believes cleaning cooking, eating and toileting areas can prevent sickness	
Yes	51%
No	5%
Unsure/Refused to answer	44%
Would clean these places more often during a pandemic	
Yes	51%
No	9%
Unsure/Refused to answer	40%
Materials used to clean these places*:	
Water only	23%
Water and soap, detergent or dishwashing liquid	20%
Disinfectants	3%
Unsure/Refused to answer	3%

*Data not available for Chiang Rai province.

### Border measures

The vast majority of respondents (90%) said they would agree to truthfully answer questions about their current health at a border crossing. More than nine in ten would truthfully tell health workers if they were feeling sick (91%) and allow health workers at the border to take their temperature (92%). Most (86%) would agree not to cross the border if sick after leaving an area with disease outbreak. Further results are presented in Table 5.

**Table 5:** Border measures (n = 773)

**Measure**	**%**
Would agree to truthfully answer questions about current health at border	
Yes	90%
No	1%
Unsure/Refused to answer	10%
Would truthfully tell health authorities at border if feeling sick, when asked	
Yes	91%
No	0%
Unsure/Refused to answer	9%
Would allow health authorities at border take temperature	
Yes	92%
No	1%
Unsure/Refused to answer	7%
Agree to not cross border if sick if leaving an outbreak area	
Yes	86%
No	1%
Unsure/Refused to answer	13%

## Discussion

We conducted a cross-sectional survey among vulnerable migrants in Northern Thailand to gain a better understanding about their perceptions of, and ability to implement, various NPIs proposed by the WHO. With the exception of border measures, to which nearly all participants reported they would be adherent, attitudes towards recommended NPIs were generally negative or uncertain: measures to reduce risk that cases transmit infection would be implemented only by a minority; perceptions towards implementing measures to reduce the risk that contacts transmit infection were somewhat better, but still a cause concern; perceived efficacy of social distancing measures was low; and, less than half of participants thought that disinfection measures could reduce the spread of influenza during a pandemic. These results demonstrate the existence of potential barriers to NPI implementation during a pandemic, suggesting that vulnerable migrants in Thailand have a limited capacity to participate in pandemic preparedness efforts.

In addition to negative perceptions towards NPIs, we also identified several other barriers: most had never worn a face mask before when sick, so are unlikely to know the correct way to do so if necessary in the future; many reported being unable to monitor their own temperatures, mainly due to not owning a thermometer and not knowing how to use a thermometer; and, most use clean water only for hand washing and disinfection, implying either a lack of knowledge about adequate disinfection or a lack of materials (e.g., soap or disinfectant).

Our results differ with those of Lau et al. [25] who found that 98.1% of people surveyed in Hong Kong surveyed would comply with quarantine measures. Only 21% and 43% of participants in this study would agree to home or hospital isolation, respectively. Social distancing could be seen as another form of quarantine. Results on social distancing in our study were somewhat comparable to a study of 813 Indians recruited from hospitals, factories, markets, and office in Udaipur Province, India [26]. The authors found that the majority of participants did not adhere to the recommended social distancing measures. We did not measure behaviour, but the negative or uncertain attitudes in our sample suggest that adherence rates would be similar. Not being able to go to work and not having access to basic necessities are two potential explanations why individuals would not want to be isolated [27].

Current results also differed from Lau et al.’s [25] sample on face mask use and hand washing. The authors comment that wearing masks is an “established practice in Hong Kong” (p.88), which might explain why the vast majority of participants had worn face masks in the past even though only about a quarter perceived them to be ‘very effective’. A similar finding was noted for hand washing. Perceptions were comparably low in our sample, but fewer participants agreed they would employ these measures.

The one area where our results matched those of Lau et al. [25] was in peoples’ willingness to tell border officials if they were feeling ill. Nearly all participants from both studies agreed they would do so. We were unable to find any comparable literature related to other border measures, or about peoples’ attitudes towards contact tracing.

There is a need to educate vulnerable groups about NPIs during inter-pandemic and pandemic periods. The WHO technical consultation on public health measures during the influenza A (H1N1) 2009 pandemic highlights the need for risk communication materials to be “adapted, tested and approved for local use ahead of time” ([1], p.23). During the A (H1N1) pandemic in Thailand risk communication was undertaken through television, radio and printed materials. However, coordination of these efforts was not always well managed and messages were sometimes inconsistent and inaccurate [10]. A better understanding about individuals’ perceptions of NPIs could help to highlight areas in which public health officials should focus risk communication and other educational activities.

Ongoing risk communication should be used to increase local knowledge. Public education campaigns have increased among migrants in Thailand since the bird flu in 2004, but knowledge levels remain low (Hickey J, Gagnon AJ, Jitthai N: Knowledge about Pandemic Influenza Preparedness among Vulnerable Migrants in Thailand, submitted). The gap between public education efforts and results highlight the inherent challenges in bringing health education to vulnerable migrant populations. Many of the migrants in this study live in remote, hard to access areas and belong to diverse cultural and linguistic groups. Migrants may also have limited experience applying recommended guidelines [28] and undocumented migrants may avoid contact with public health officials due to fear of deportation. For risk communication to be effective, it must address these challenges and incorporate a component designed to improve people’s perceptions of NPIs. Widespread implementation of NPIs will be unlikely if public perceptions remain low [11,12].

Future research efforts should continue to assess the perceptions and ability of diverse populations relating to implementation of NPIs. These data could provide valuable information to public health agencies with regard to planning for future outbreaks and pandemics and assessing risk communication and public education activities. In the current inter-pandemic period, it would also be beneficial to develop and test measures to improve perceptions towards and understanding of NPIs, particularly among potentially vulnerable populations. Ongoing efforts to systematically assess and standardize public education campaigns and risk communications for consistency and effect should also continue, as should the development of culturally and linguistically appropriate materials.

This study has several limitations. Translation of some concepts (e.g., pandemic) into migrant languages was sometimes difficult. This difficulty was addressed by working with MCHWs to determine acceptable translations. Because this was a cross-sectional study with data collected at one time point only we were not able to measure potential changes in behaviour resulting from NPI recommendations. As such we are not able to say with certainty that our results are associated with decreased capacity to enact NPIs, though based on the literature presented it seems likely that this would be the case. Our results provide a baseline that may be useful in assessing future public education efforts.

External validity in this study was reinforced by random sampling of households and high participation rates. Validity may be threatened by our decision to request an individual volunteer from each randomly selected household. Census data is not available for this population, but comparison with socio-demographic data from relevant studies [7,29] suggests that we obtained a representative sample. We were able to enroll a volunteer from each household that was sampled, but despite attempts to ensure completeness, 28 participants had to be excluded from data analysis due to missing answers. Internal validity was strengthened by the incorporation of existing questionnaires and concepts into an adapted tool, expert review of the adapted tool, rigorous translation, and extensive cultural review and pretesting of the final instrument. We did not conduct a factor analysis due to time/resource constraints.

## Conclusion

WHO pandemic guidance documents propose that individuals have a role to play in pandemic preparedness. However, if these individuals do not know how to fulfill that role or do not believe that certain interventions will be effective, they are unlikely to take part in the role that has been proscribed to them. Results from the current study suggest that vulnerable migrants in Thailand have a limited capacity to participate in pandemic preparedness efforts due to negative or uncertain attitudes towards NPI effectiveness and an inability to enact certain NPIs. This limited capacity likely puts this population of migrants at risk for contracting and transmitting influenza during periods of outbreak and pandemic. Current results highlight the need for ongoing, culturally-appropriate, multi-lingual risk communication and public health education. Research into the appropriate use of risk communication during inter-pandemic and pandemic periods, combined with ongoing education at the community level, could potentially strengthen individuals’ capacity to participate in pandemic preparedness efforts.

## Abbreviations

IOM: International Organization for Migration; MCHW: Migrant Community Health Worker; MOPH: Ministry of Public Health; NPI: Non-pharmaceutical intervention; THB: Thai Baht; USD: United States Dollars; WHO: World Health Organization.

## Competing interests

The authors declare that they have no competing interests.

## Authors’ contributions

JH contributed to project development, led implementation, and was responsible for leading data analysis and manuscript publication. AG and NJ contributed to project development, supported implementation, and guided data analysis and manuscript drafting. All authors read and approved the final manuscript.

## Authors’ information

Nigoon Jitthai is the former employee of the International Organization for Migration (IOM).

## Pre-publication history

The pre-publication history for this paper can be accessed here:

http://www.biomedcentral.com/1471-2458/14/665/prepub
